# Using low levels of stochastic vestibular stimulation to improve locomotor stability

**DOI:** 10.3389/fnsys.2015.00117

**Published:** 2015-08-24

**Authors:** Ajitkumar P. Mulavara, Igor S. Kofman, Yiri E. De Dios, Chris Miller, Brian T. Peters, Rahul Goel, Raquel Galvan-Garza, Jacob J. Bloomberg

**Affiliations:** ^1^Universities Space Research Association, Houston, TXUSA; ^2^Wyle Science Technology and Engineering Group, Houston, TXUSA; ^3^University of Houston, Houston, TXUSA; ^4^Massachusetts Institute of Technology, Cambridge, MAUSA; ^5^Johnson Space Center, National Aeronautics and Space Administration, Houston, TXUSA

**Keywords:** stochastic resonance, balance control, vestibular stimulation, stochastic electrical stimulation, body motion threshold, perceptual motion threshold

## Abstract

Low levels of bipolar binaural white noise based imperceptible stochastic electrical stimulation to the vestibular system (stochastic vestibular stimulation, SVS) have been shown to improve stability during balance tasks in normal, healthy subjects by facilitating enhanced information transfer using stochastic resonance (SR) principles. We hypothesize that detection of time-critical sub-threshold sensory signals using low levels of bipolar binaural SVS based on SR principles will help improve stability of walking during support surface perturbations. In the current study 13 healthy subjects were exposed to short continuous support surface perturbations for 60 s while walking on a treadmill and simultaneously viewing perceptually matched linear optic flow. Low levels of bipolar binaural white noise based SVS were applied to the vestibular organs. Multiple trials of the treadmill locomotion test were performed with stimulation current levels varying in the range of 0–1500 μA, randomized across trials. The results show that subjects significantly improved their walking stability during support surface perturbations at stimulation levels with peak amplitude predominantly in the range of 100–500 μA consistent with the SR phenomenon. Additionally, objective perceptual motion thresholds were measured separately as estimates of internal noise while subjects sat on a chair with their eyes closed and received 1 Hz bipolar binaural sinusoidal electrical stimuli. The optimal improvement in walking stability was achieved on average with peak stimulation amplitudes of approximately 35% of perceptual motion threshold. This study shows the effectiveness of using low imperceptible levels of SVS to improve dynamic stability during walking on a laterally oscillating treadmill via the SR phenomenon.

## Introduction

In general terms, stochastic resonance (SR) can be thought of simply as “noise benefit” by increasing information transfer in the presence of non-zero level of noise (for reviews, see Collins et al., 2003; Moss et al., 2004; McDonnell and Abbott, 2009; Aihara et al., 2010). SR has been observed in human hearing (Jaramillo and Wiesenfeld, 1998; Zeng et al., 2000; Ward et al., 2002) and has been identified as an important component in cochlear coding strategy (Morse and Evans, 1996). The presence of stochastic noise to sensory input has been shown to improve: visual contrast sensitivity and detection (Simonotto et al., 1997; Ward et al., 2002); the degree of association between the heart rate responses and weak periodic oscillatory variation in central venous pressure (Soma et al., 2003); letter recognition (Piana et al., 2000); perception of ambiguous figures (Riani and Simonotto, 1994); and visual depth perception (Ditzinger et al., 2000). SR in tactile sensation has been demonstrated in the response to weak mechanical stimuli (Collins et al., 1996a,b, 1997; Ivey et al., 1998; Richardson et al., 1998). The application of mechanical noise to the feet has been shown to improve balance control through the reduction of sway in young and elderly subjects (Priplata et al., 2002, 2003), in patients with diabetes and stroke (Priplata et al., 2006) and patients with functional ankle joint instabilities (Ross and Guskiewicz, 2006; Ross et al., 2013). Similarly, balance improvement has been demonstrated with electrical noise applied to the back of the knee (Gravelle et al., 2002). Vibratory noise applied to the fingertip also enhanced balance performance based on SR phenomenon (Magalhaes and Kohn, 2011). These same authors have also shown that the application of imperceptible electrical noise to the triceps surae during a seated task reduced force fluctuations in a force matching task of isometric plantar flexion force which were correlated to subsequent reductions in postural sway during quiet stance based on SR phenomenon (Magalhaes and Kohn, 2012). There have been a few studies that showed the effectiveness of applying sub-sensory vibratory noise to the soles of the feet during over-ground walking comparing elderly population with young control subjects (Galica et al., 2009). In a follow-up study, this group also showed the effectiveness of applying sub-sensory vibratory noise to the soles of the feet during treadmill walking in a set of control subjects (Stephen et al., 2012).

Over the past century, transcutaneous binaural vestibular electrical stimulation (Galvanic vestibular stimulation, GVS) applied across the mastoid bones at amplitudes ranging from 0.5–5 mA has been used to study and understand the function of the vestibular system while studying eye movement responses, as well as by eliciting disruption of balance control during standing and walking (for review, see Hlavacka and Njiokiktjien, 1985; Fitzpatrick and Day, 2004; MacDougall et al., 2006; Moore et al., 2006; Cohen et al., 2011). In all of these studies the electrical stimulation profile for GVS was either a direct current (DC) or slowly varying DC (zero mean) applied to the mastoids. Recently, repeated application of high magnitudes of slowly varying GVS (peak magnitudes of 5 mA) over repeated sessions have been shown to improve postural stability to unexpected perturbation type stimulus and have been hypothesized to work by training the sensorimotor integration process to not use vestibular information during the performance of these tasks (Dilda et al., 2014; Moore et al., 2015). The technique of applying stochastic vestibular stimulation (SVS) have also been used to investigate the motor responses elicited by the vestibular system (Dakin et al., 2007) as well as the disruption of control of nominal body responses in posture, and balance to unpredictable vestibular perturbations (Fitzpatrick et al., 1996; Pavlik et al., 1999). Studies using SVS have designed the stimulus profile with a white noise stimulus signal having power equal at all frequencies or a colored noise stimulus signal having a power spectrum that decreases with increasing frequency (Fitzpatrick et al., 1996; Pavlik et al., 1999; Soma et al., 2003).

Several studies have also shown performance improvement with SVS based on SR by improving long-term heart rate dynamics and motor responsiveness in clinical populations with multisystem atrophy and Parkinson’s disease (Yamamoto et al., 2005) and specifically to improve postural balance performance in PD patients (Pal et al., 2009; Samoudi et al., 2015). Low imperceptible levels of SVS have been shown to improve stability during balance tasks in normal, healthy subjects (Mulavara et al., 2011, 2012). All these studies showing improvement in function using SVS have used imperceptible peak current levels of less than 1 mA that were below subjects’ threshold of detection. The aim of the current study was to investigate the effectiveness of using low imperceptible levels of SVS to improve dynamic stability during walking on a laterally oscillating treadmill via the SR phenomenon.

## Materials and Methods

### Participants

Thirteen subjects, with mean ± SD age 40.8 ± 10.1 years, height 175.5 ± 11.8 cm, and weight 77.5 ± 17.1 kg, were recruited from the Human Test Subject Facility at NASA Johnson Space Center (JSC) in Houston, TX, USA. All of these subjects had passed the equivalent of an Air Force Class III physical examination within I year of beginning participation in this study. The Institutional Review Board at NASA JSC approved the experimental protocol. All subjects gave a written informed consent before their participation in the study.

### Procedures

#### Electrode Placement

For each subject, the 5 cm × 10 cm electrodes (Axelgaard Manufacturing, Fallbrook, CA, USA) were centered over the mastoid processes on both sides using methods described in our previous paper and described briefly herein (Mulavara et al., 2011). The electrode site skin surface was cleaned and dried, and a layer of electrode gel was applied on the electrodes before placing them on the skin surface centered on the mastoid bone. Electrodes with soft pads placed over them were held in place by using an elastic strap to achieve the application of a uniform current density stimulus distribution across the wide area of the skin-electrode interface without adding constrains to head movements. This methodology was adopted to minimize irritation at the electrode site during the delivery of electrical stimulus. The impedance between the electrodes was confirmed to be less than 1 kΩ.

#### Treadmill Locomotion

**FIGURE 1:**
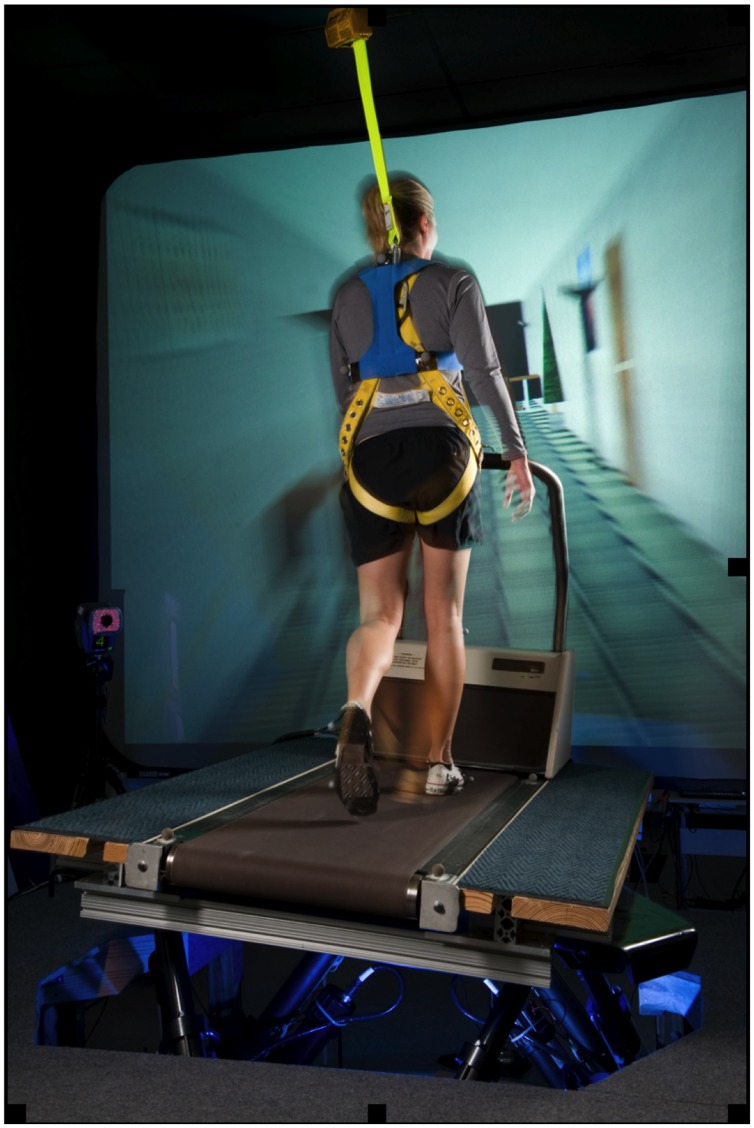
**An exemplar subject performing the walking task on a treadmill mounted on a six degree of freedom motion base that provided continuous, sinusoidal, lateral motion of the support surface.** Subjects were simultaneously viewing perceptually matched linear optic flow while walking on the treadmill.

The subjects walked on a treadmill while simultaneously viewing perceptually matched linear optic flow and while the support surface was continuously oscillated with a sinusoidal lateral motion (**Figure 1**) as detailed in previous studies (Brady et al., 2009; Peters et al., 2012). A standard treadmill (Quinton Model Q55, A-H-Robins Company, Philadelphia, PA, USA) was mounted on a commercially available, six degree-of-freedom (6 DoF) electric motion base (Moog, East Aurora, NY, USA). Movement of the motion base was a 0.254 m peak-to-peak continuous lateral, sinusoidal translation at 0.33 Hz. Subjects walked at a fixed walking speed at 1.12 m/s throughout all the trials. Each trial was 60 s long. A computer-generated virtual hallway scene was back-projected onto a 3.6 m × 2.6 m screen positioned 2 m in front of participants and moving linearly at perceptually matched speeds. The hallway interior constantly translated along the anterior posterior body axis along with movement of the motion base so that the participants perceived walking down a hallway at a fixed rate that matched their speed of walking on the laterally moving surface. Participants were asked to walk on the treadmill as naturally as possible, look straight ahead, and refrain from handrail use unless they deemed it necessary for safety. Test operators did not initiate conversation during the test.

Balance performance during locomotion was measured by using an inertial measurement unit (IMU) motion sensor (MTx, Xsens North America Inc., Los Angeles, CA, USA) placed on a vest tightly worn by the subjects with the IMU at the level of C7 vertebrae on the trunk segment. An additional IMU was fixed to the treadmill base to enable the measurement of relative motion of the subject trunk segment with respect to the moving support surface. The IMU data were sampled at 100 Hz. In addition, heel strike event timing data during walking were measured using footswitches (Motion Lab Systems; Baton Rouge, LA, USA) affixed to shoe soles at the heels. These data were sampled at 1000 Hz. Custom LabVIEW (National Instruments; Austin, TX, USA) program was used to acquire the motion sensors and footswitch data streams synchronously.

A portable constant-current stimulator with subject isolation was used to deliver bipolar binaural SVS. The stimulator, capable of delivery of current in ±5 mA range to a load of up to 10 kΩ, was powered using a 3.7 V battery pack and was operated in a standalone mode with the stimulation profiles stored on its internal flash memory card. The signature of SR is a bell-shaped relationship between the detection performance and noise level such that when the input noise added to a system is too low, the system does not experience any performance gains. If the noise level is too high, the system may become saturated, rendering it unable to distinguish between signal and noise and again performance is degraded. The optimal noise level is that which is associated with some optimal system output occurring at the peak of the trademark bell-shaped SR curve (Moss et al., 2004). This type of improved system behavior due to noise is the characteristic manifestation of SR. Therefore, eleven stimulation amplitude levels were tested with the peak current values of 0, 100, 200, 300, 400, 500, 700, 900, 1100, 1300, 1500 μA.

Bipolar SVS signals, similar to those used in our previous study (Mulavara et al., 2011) were generated using a Gaussian white noise pattern generator and then filtered using a 10th order low-pass Butterworth filter with the cutoff frequency at 30 Hz using LabVIEW version 9.0 (National Instruments, Austin, TX, USA). In our previous study, we applied a wide range of imperceptible noise levels to the vestibular system of subjects during a standing balance task and compared the effects of SVS on postural balance using both colored noise signal (generated using a stochastic process described as a relaxator driven by white noise as described in Pavlik et al., 1999) in the narrow band 1–2 Hz and a Gaussian white noise signal in the wide band 0–30 Hz frequency ranges (See Figure 2 in Mulavara et al., 2011). The two signals were found to be similarly effective in improving balance through a reduction of sway. It was also reasoned with supporting evidence from other studies (Dakin et al., 2007; Songer and Eatock, 2013) that the wideband range of 0–30 Hz improved performance by stimulating vestibular hair cells (VHCs) that affect posture (lower frequency sensitivity) and evoke vestibulo-myogenic response in the lower limbs (higher frequency sensitivity). Therefore, in this study a Gaussian white noise signal in the wideband 0–30 Hz frequency range was used in the stimulation profile. The fully generated SVS signal was checked for zero mean (±1%) and variability using the root mean square (RMS) to be 26 μA RMS/100 μA peak (±5%). Each trial was 60 s long and composed of two periods: 30-s zero amplitude stimulus current baseline period followed by a 30-s stimulation period with the peak current values for each of the 11 trials varying from 0–1500 μA as noted above. The order of presentation of stimulation amplitude levels was randomized across the 11 trials for each subject. The trials with zero-amplitude current during the stimulus period were considered control trials while all other trials were the experimental trials.

#### Thresholding Task

The thresholding task was designed to identify the level of electrical vestibular stimulation at which subjects were able to discern motion induced by the stimulation. Subjects sat on a wooden stool with backrest, with their feet placed on the footrest, and held a gamepad (Logitech Gamepad F310, Lausanne, Switzerland). Subjects were facing forward with eyes closed. Subjects were instructed to report any perceived motion sensation by moving one of the joysticks with their dominant hand. The exact instruction given to each subject was: “Use your dominant hand to push a joystick depending upon the direction of the motion sensation. Make sure to do it as long as you feel the sensation.” Data were collected from the joystick at 1000 Hz. The thresholding task was performed on a different day after the session during which treadmill locomotion task were performed.

We were interested in investigating the relationship between perceptual motion threshold amplitude and the peak stimulation level that resulted in optimal walking performance. Previously, in studies investigating effects of electrical vestibular stimulation, a periodic stimulus has been used to identify vestibular sensory threshold, which is usually the intensity at which the experimenter observes body sway at the input frequency (Pavlik et al., 1999; Bent et al., 2004; Samoudi et al., 2015). In some other studies a stochastic signal was used to define threshold based on subjects’ perception of irritation on the skin (Yamamoto et al., 2005; Utz et al., 2011). Hence, two different types of stimulation signals were used for perceptual motion threshold estimation in this study: 1 Hz sinusoidal signal and 0–30 Hz stochastic signal. The 0–30 Hz stochastic signal was the same as that used during the walking task. However, subjects reported perceiving no induced motion when receiving stochastic signal stimulation with peak current amplitudes between 0 and 1500 μA. This may be attributed to the stochastic nature of the electrical stimulation resulting in subjects’ inability to perceive or delineate motion in a specific direction. Hence, the 1 Hz sinusoidal electrical stimulation signal was chosen for motion threshold determination in this study. In general the stimulus profile consisted of 15 s periods with the 1 Hz sinusoidal stimulation signals, interspersed with 20 s periods of no stimulation. The different current peak amplitudes used were 100, 200, 300, 400, 500, 600, 700, 900, 1100, 1300, 1500 μA. The order of the stimulation levels, within the profile, was randomized for all subjects. The total duration for this task was 407 s.

### Data Analysis

#### Walking Task

In previous studies investigating human walking for a period of 20 min on a laterally oscillated treadmill, subjects showed considerable variability in gait pattern during the initial period after the onset of perturbation for up to the first 5 min before a stable gait pattern emerged (for details, see Brady et al., 2009; Figure 2 in Peters et al., 2012). In our current study, we were specifically interested in the efficacy of SVS on the stability of walking during the acute phase of 1 min after the onset of perturbation. Further, other studies have shown the usefulness of variability in gait parameters such as gait cycle time to differentiate stability during walking between the young and elderly populations (Menz et al., 2003) as well as in identifying falls in patients with cerebellar ataxia (Schniepp et al., 2014). In this paper, the variability in gait is taken as a measure of the central nervous systems’ (CNS) ability to regulate and maintain a stable gait pattern and inferred to measure dynamic stability under imposed perturbation conditions (Hausdorff, 2005).

Linear acceleration and angular velocities were measured by the IMU’s attached to a vest worn tightly by the subject to measure their trunk motion along with an IMU attached to the moving base platform. Kinematic data were low-pass filtered with a recursive second-order Butterworth filter at cut-off frequencies of 10 Hz. At the start of the protocol, with the base and treadmill stationary, subjects were asked to stand such that their trunk and base coordinate system were set parallel to a global reference frame with +Z always directed in the superior direction, +X always directed toward the front and +Y-axis in the mediolateral direction to form a right handed coordinate system. During the walking trials the trunk IMU data were transformed into the base coordinate reference frame to enable the calculation of the relative acceleration and angular velocities in the three directions in the base coordinate reference frame using the expression (Shigley and Uicker, 1981):

$$A_{t/b}=A_{t}-A_{b}-A_{tb}^{C}$$

$$A_{tb}^{C}=2w_{b}\times V_{t/b}$$

$$V_{t/b}=V_{t}-V_{b}$$

where, A_t_ is the linear acceleration of the trunk, A_b_ is the linear acceleration of the base, A_t/b_ is the relative acceleration of the trunk with respect to the base; $A_{tb}^{C}$ is the coriolis term; *V*_t_ is the linear velocity of the trunk, V _b_ is the linear velocity of the base, V_t/b_ is the relative linear velocity of the torso with respect to the base; w_b_ is the angular velocity of the base. The RMS of the relative trunk linear accelerations: fore-aft (Tax), mediolateral (Tay) and superior-inferior (Taz) directions and relative trunk angular velocities: roll (Trv), pitch (Tpv), and yaw (Tyv) directions for the middle 25 s for each of the baseline and stimulus periods were calculated for each trial.

Custom MATLAB programs were used to analyze the footswitch data as in our previous studies (Batson et al., 2011; Brady et al., 2012). Data from the middle 25 s for each of the baseline and stimulus periods were used for further analysis to remove end effects for each trial yielding up to 21 gait cycles for both periods for each trial. Gait cycle time was determined based on right-foot heel strikes. In addition to the kinematic parameters, walking stability was also determined by monitoring variability of gait cycle timing using a coefficient of variation parameter – Gait cycle time variability (GCV). GCV was defined as the percentage of the ratio of the SD of the gait cycle time normalized to its average over the 21 gait cycles during each baseline and stimulation period for each trial.

Methods similar to those used in our previous paper (Mulavara et al., 2011) were used to evaluate improvements in balance performance. Thus, for each subject, the ratio of stimulus period to the baseline period for all trials was calculated for each of the seven parameters. A cost function was estimated as the sum of these ratios across all the seven parameters for each trial. A number of studies that have shown improvement of function using the SR phenomenon have limited the magnitude of stimulus used at or below the subject threshold (Yamamoto et al., 2005; Wilkinson et al., 2008; Samoudi et al., 2015). Further, other studies (Dilda et al., 2014; Moore et al., 2015) have shown that balance function can be improved by repeatedly exposing subjects to supra-threshold sum of sine electrical stimulation of the vestibular system by the process of desensitization of the sensorimotor integration process to ignore inputs from the vestibular system. Therefore, for each subject, the optimal stimulus amplitude (i.e., optimal trial) were determined as the one at which the value of the cost function was the lowest among the trials with non-zero stimulation periods with amplitudes less than the subjects’ perceptual threshold. Hence, the cost function for the optimal trial would be indicative of the highest improvement in walking stability across the seven parameters in the stimulus period relative to the corresponding baseline period across the experimental trials with non-zero peak stimulus current levels. We also identified subjects as responsive, i.e., exhibit improvement in balance performance in experimental trial compared to corresponding control trial, if the value of this cost function within any of their trials with stimulus amplitudes less than the subjects’ perceptual threshold was less than that calculated for their control trials.

We compared data for each of the seven parameters from all subjects between the control and optimal trials using repeated measures analysis of variance with two within-subject factors: Period (two levels—baseline and stimulus), and Trials (two levels—control and optimal) at a significance level of 0.05. In addition, for each parameter separate paired *t*-tests were used to compare the control and optimal trials, using their ratios for stimulus with respect to baseline periods, at a significance level of 0.05.

#### Thresholding Task

Raw joystick data were used in data analysis. During pilot testing for this study, subjects’ reports of perceptual responses of motion during applied stimulus were predominantly in the mediolateral direction. Hence we limited our analyses of joystick to the mediolateral direction only. For the joystick data, percentage time of “perceived motion reported by the subject” for each stimulation and baseline period was calculated. Joystick movement was interpreted as “perceived motion reported by the subject,” when the signal amplitude exceeded 0.05 V (full-scale movement recorded in 0–5 V range). The percentage time at each stimulation and baseline level for perceptual motion detection was normalized with respect to the largest value across all levels of stimulation. While the primary criterion was the decision whether the subject detected the signal or not, there were only two possible outcomes, “yes” (1) or “no” (0). Hence, a binomial distribution function was fit to the data using a generalized linear model and a logit link function, which is very common in psychophysical studies (Treutwein, 1995). Threshold was defined as the amplitude of stimulation at the point of subjective equality, at which there is a 50% chance of motion detection (Treutwein, 1995; Moss et al., 2004). A linear regression analysis was performed to examine the relationship between the peak stimulus amplitude at which optimal balance performance was determined and perceptual threshold amplitude using a significance level of 0.05.

## Results

**Table 1** describes the subjects’ demographics, optimal value of the peak current amplitudes for the SVS for optimal trials, the threshold amplitude of perceptual motion for all 13 subjects, whether the subject was responsive during their optimal trials with respect to the control trials and the trial numbers at which their optimal performance was determined. Subjects did not report unpleasant symptoms during or after the stimulation trials during the treadmill locomotion task. Also, subjects did not report any awareness of being stimulated during these trials.

**Table 1:** Subjects’ demographics, optimal value of the peak current amplitudes for the stochastic vestibular stimulation (SVS) for optimal trials, the threshold amplitude of perceptual motion, whether the subject was responsive during their optimal trials with respect to the control trials and the trial number at which optimal performance was exhibited out of a maximum of 11 trials.

Serial No.	Age	Gender	Height (cm)	Weight (kg)	Optimal current peak amplitude (μA)	Perceptual threshold peak current amplitude (μA)	Responder?	Trial No.
1	40	M	180	95	300	395	Y	2
2	60	M	185	112	200	1380	Y	8
3	32	M	190	90	100	650	Y	11
4	45	M	183	88	500	1405	Y	11
5	54	M	185	80.7	200	Not Available	Y	5
6	35	M	187	86	200	960	Y	1
7	33	M	177	73	500	940	Y	8
8	54	F	166	60	100	1085	Y	7
9	32	M	185	82	100	1185		6
10	40	F	163	62	500	500	Y	9
11	26	F	156.5	55.5	200	355		8
12	36	F	165	68.5	100	600	Y	10
13	43	F	159	55	100	730		4

**FIGURE 2:**
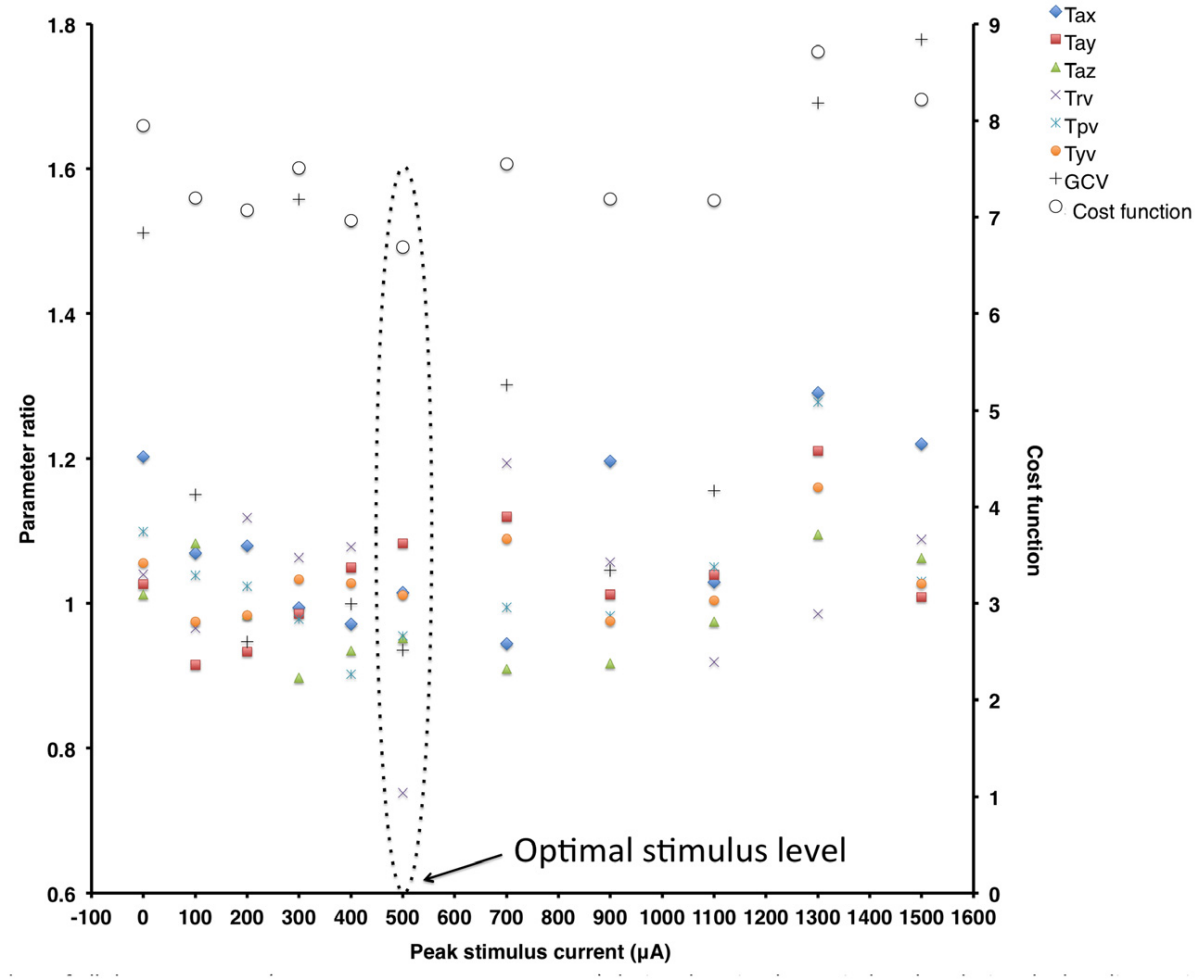
**Ratio data of all the parameters [Tax, Tay, Taz, Trv, Tpv, Tyv, Gait cycle time variability (GCV)] during the stimulus period to that during the baseline period (plotted on the primary *y*-axis on the left) and the cost function value (plotted on the secondary *y*-axis on the right) at different peak stimulus amplitudes for a typical subject**. Note that for this subject, locomotor balance performance improved at the peak stimulus amplitude of 500 μA.

**Figure 2** shows ratio data of all the measures (Tax, Tay, Taz, Trv, Tpv, Tyv, and GCV) during the stimulus period to that during the baseline period (plotted on the primary *y*-axis on the left) and the cost function value (plotted on the secondary *y*-axis on the right) at different peak stimulus amplitudes for a typical subject. These data show that this subject is responsive to SVS (i.e., shows improvement in an experimental trial relative to the control trial) showing peak improvement at a non-zero peak SVS current level that gradually reduces at higher current levels consistent with SR phenomenon. The optimal response for this subject to improve stability during walking on the perturbed surface was at the stimulation level with peak current amplitude of 500 μA, which was approximately at this subjects’ perceptual motion threshold.

**FIGURE 3:**
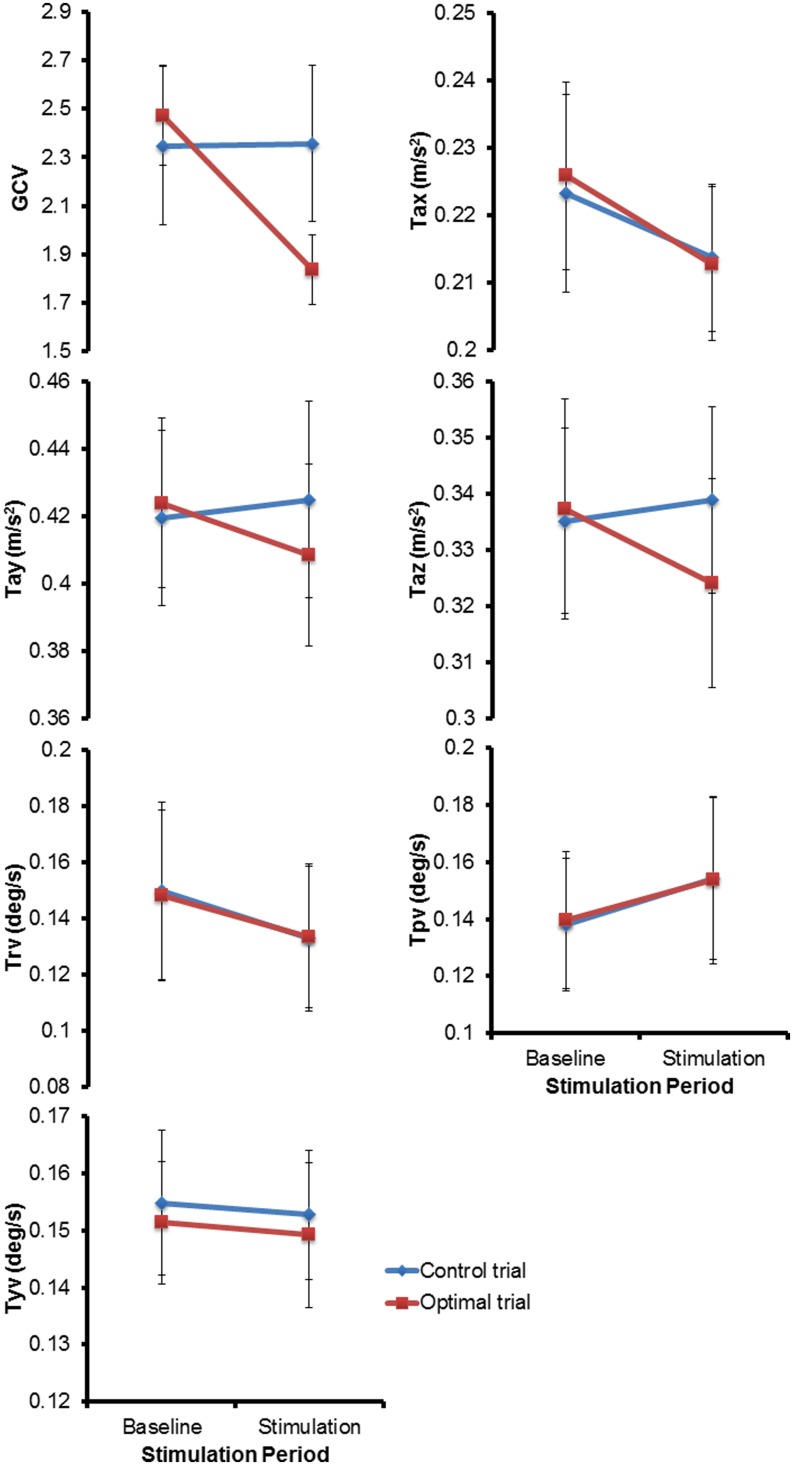
**The mean (± 1 SEM) values across all subjects (*n* = 13) for all the seven parameters of interest during the two periods (baseline and stimulus) of the control and optimal trials**.

**FIGURE 4:**
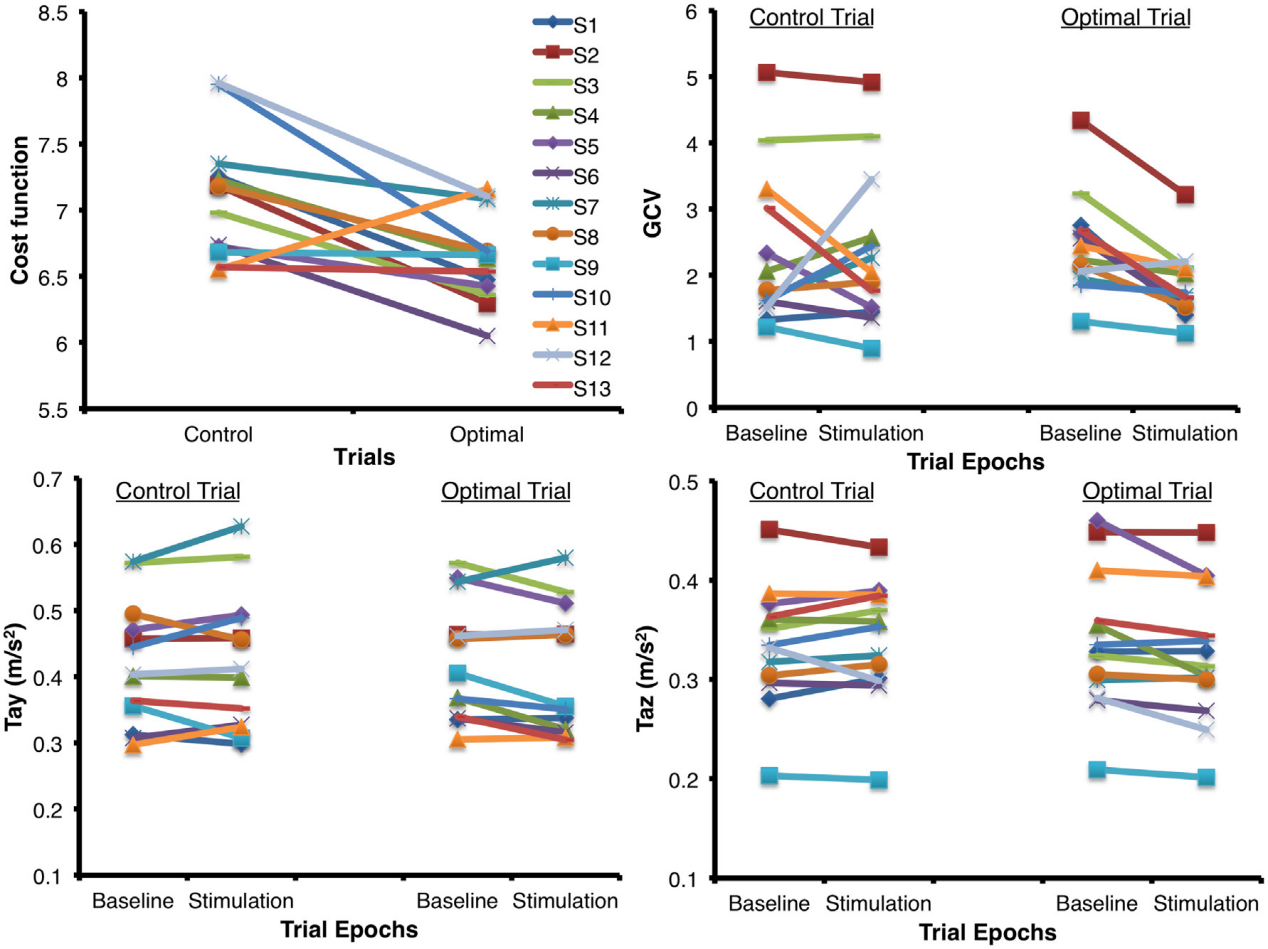
**Individual values for the cost function value calculated as the sum of ratios for the stimulus period to the baseline period across the seven parameters for the control and optimal trials as well as the individual values for the parameters GCV, Tay, and Taz that showed significant interactions between the factors of Period and Trial (*n* = 13) during the two periods (baseline and stimulus) of the control and optimal trials**. Please note that the subject in **Figure 2** is subject#10 in this figure.

**Figure 3** shows the mean (±1 SEM) values across all subjects for all the seven variables of interest (*n* = 13) during the two baseline and stimulus periods of the control and optimal trials. The repeated measures ANOVA with two within-subject factors: Period (two levels – baseline, stimulus), and Trial (two levels – control and optimal) on each of the parameters showed that the within-subject factor Trial was significant only for Tax [*F*(1,12) = 6.329, *p* = 0.027] while the interaction Trial ^∗^ Period was significant for the parameters GCV [*F*(1,12) = 10.020, *p* = 0.008], Tay [*F*(1,12) = 5.212, *p* = 0.041] and Taz [*F*(1,12) = 8.266, *p* = 0.014]. None of the other factors were significant for any of the parameters (*p* > 0.05). Larger decreases in GCV, Tay and Taz parameters in the stimulation periods relative to the baseline periods for the optimal trials compared to that for the control trials resulted in the significant interaction of Trial ^∗^ Period. Paired student *t*-test between the control and optimal trials, comparing the percent change in values for the stimulus period with respect to their corresponding baseline periods, revealed a significant difference between the trials for GCV (*p* = 0.011), Tay (*p* = 0.040), and Taz (*p* = 0.004) across all subjects (*n* = 13). Overall, these results show that at optimal levels of SVS this group of normal healthy subjects significantly improved their walking stability and extends our previous results (Mulavara et al., 2011) that showed improvement with SVS in postural control during a standing task consistent with SR phenomenon. The peak amplitude of stimulus during optimal trials for improving walking stability was predominantly in the range of 100–500 μA (See **Table 1**). **Figure 4** shows individual values for the cost function value calculated as the sum of ratios for the stimulus period to the baseline period across the seven parameters as well as for the parameters GCV, Tay, and Taz for the control and optimal trials during the two baseline and stimulus periods of the control and optimal trials for each individual subject (*n* = 13). Given the significant results across all subjects, it is important to point out that these analyses also showed that only a subset of subjects (10 out of 13) were responsive to the SVS, showing an improvement in the optimal trials with respect to control trials as seen in the **Figure 4** showing the cost function for individual subjects. Two subjects out of the 13 subjects showed greater improvement in walking performance at supra-threshold current amplitudes than that shown at their individual optimal trial (one non-responder at 110%, and one responder at 127% of their individual perceptual thresholds). Overall, SVS resulted in an average improvement (percentage change of ratio between the stimulus period normalized to baseline period in the optimal trials with respect to that for the control trials), in the range of 3–21% across the GCV, Tay, and Taz parameters.

We were specifically interested in using the perceptual threshold amplitude for predicting the stimulation level that would result in optimal walking performance. We used the threshold estimates obtained from the perceptual measure to compare with the optimal trial peak current levels using available data from 12 of 13 subjects who completed their walking assessments. However, optimal trial peak current levels showed no significant correlations with the perceptual threshold amplitude (*p* > 0.05). The average peak current amplitude of SVS stimulation that resulted in improved balance performance in terms of percentage of perceptual thresholds was estimated to be 35%.

## Discussion

In this study, we investigated the efficacy of imperceptible white noise SVS on locomotion balance performance while walking on a laterally oscillating treadmill. The study demonstrates that low imperceptible levels of white noise SVS significantly improves walking stability as measured by the reduction in variability of gait cycle time and the trunk accelerations in the plane of perturbation. The peak current amplitude of optimal stimulus for improving walking performance was predominantly in the range of 100–500 μA. The behavior of the outcome variable with peak of performance at a non-zero noise application and then a reduction of performance at higher noise levels is consistent with the SR phenomenon in normal healthy subjects.

Walking stability was measured during both the baseline period in which 0 μA of current was applied, and the corresponding paired stimulus period when different peak amplitudes of stimulation current was applied, in the same trial. This helped track subjects’ performance in each of the experimental trials during stimulus presentation relative to their baseline ability to control their walking stability on the challenging oscillating support surface in comparison to the control trial when zero amplitude stimuli was presented during both periods. There was an average (1 SEM) increase in GCV by 7.4 (12.8) %, in RMS of Tay by 1.1 (2.0) %, and RMS of Taz by 1.2 (1.3) % between the baseline and stimulation periods for the control trials with zero amplitude of stimulus application. The amplitude variation in the GCV, Tay, and Taz values across subjects (**Figure 4**) in transition from the baseline to stimulus periods during the control trials, when no current was presented for the entire duration of the trial, indicates that the inherent variability of the walking stability that increased or remained the same across subjects as they were adapting to the support surface characteristics within the trial. In contrast, SVS resulted in an average improvement (i.e., decrease) in GCV by 23.7 (4.6) %, in RMS of Tay by 3.8 (1.7) %, and in RMS of Taz by 4.2 (1.4) % during the stimulation periods with respect to their corresponding baseline periods for the optimal trials. Hence, given the baseline variations when no current was applied during the control trials, our results indicate that for the optimal trials of walking on a continuously oscillating surface, the GCV, RMS of Tay, and RMS of Taz were significantly reduced. This indicated an improvement in walking stability on a laterally oscillating support surface during the stimulation period relative to the baseline period, when compared with control trials, as a result of the application of the low levels of electrical stimulation to the vestibular system.

Results showed a greater significant reduction in variability of the gait cycle timing and a relatively smaller but significant reduction in the variability of trunk accelerations in the plane of perturbation (Tay and Taz) from baseline to stimulus periods for the optimal trial with respect to that seen for the control trials (see **Figure 3**). Several studies have demonstrated the influence of the vestibular system on the control of lower body and spatial orientation during walking (Bent et al., 2004; Moore et al., 2006). The vestibular system not only helps in monitoring and providing information that is utilized to stabilize the body during unstable conditions during standing and walking but also in providing direct feed-forward mechanisms to plan for forward progression during walking such as correct foot placements to improve stability (Bent et al., 2004). Moore et al. (2006) have shown that disrupting the functioning of the vestibular system during walking specifically causes disruption in the control of head-trunk coordination and mobility performance. Bent et al. (2004) have specifically shown in their experiments that disrupting the functioning of the vestibular system resulted in a greater change in foot placement than on upper body movement control during walking. These results from earlier studies thus may explain the smaller but significant improvement seen in the reduction of the upper body movement variability in the plane of perturbation and the relatively larger significant improvements seen in the reduction of variability in gait cycle timing with the application of low level amplitude SVS while walking on a laterally oscillating surface.

Three out of the 13 subjects were not responsive to SVS during the optimal trials and did not show relative improvement with respect to control trials as a result of the application of low-levels of SVS in walking performance. However, these three non-responding subjects also showed that during their respective control trials when no stimulation was applied the GCV had already decreased (i.e., improved stability) by 35.4% (1 SEM = 4.4%) while in contrast across the responding subjects there was an increase in GCV by 26.4% (1 SEM = 14.4%) from the baseline to the stimulation period. However, during the optimal trials GCV for the non-responding subjects decreased only by 21.8% (1 SEM = 7.9%) while the responding subjects showed a decrement of 22.4% (1 SEM = 6.1%) from the baseline to the stimulation period. We infer that these three subjects, who did not show a relatively greater improvement during their optimal trials and were already performing better during their control trials, may have increased preferences for the weighting of sensory contributions from other sensory inputs such as vision and proprioception in the sensorimotor integration process for locomotor control during perturbations (Peterka, 2002; Peterka and Loughlin, 2004). Comparatively, both the group of responding subjects and the three non-responding subjects showed an increase of 1.86% (1 SEM = 2.0%) and 2.6% (1 SEM = 6.5%) in RMS of Tay, respectively, and 0.95% (1 SEM = 5.5%) and 1.12% (1 SEM = 4.1%) in RMS of Taz, respectively, from the baseline to the stimulation period during their control trials when no stimulation was applied. Both the group of responding subjects and the three non-responding subjects showed a decrease of 2.3% (1 SEM = 2.0%) and 7.3% (1 SEM = 4.2%) in RMS of Tay, respectively, and 3.6% (1 SEM = 1.9%) and 3.1% (1 SEM = 0.84%) in RMS of Taz, respectively, from the baseline to the stimulation period during their optimal trials. These relatively modest and equal improvements in stability of gait pattern during the optimal trials for the trunk acceleration do show that SVS did help improve trunk movement stability during perturbed locomotion. Our data also showed that two subjects showed improved walking stability at supra threshold levels greater than that seen during their optimal trials below their perceptual threshold amplitudes. Results for these two subjects offers support to the explanation of subjects’ ability to down-weight vestibular information specifically when exposed to supra threshold levels of stimulation that disrupt the vestibular sensory transduction process (Dilda et al., 2014).

One of the objectives in this study was to be able to customize subjects’ optimal stimulation levels as a function of their objectively measured stimulation-induced motion threshold. This approach can maximize improvements in balance performance effects for individual subjects as compared to other approaches such as using perithreshold levels of stimulation as used in the Samoudi et al. (2015) study. We propose an objective method to determine a robust estimate of subjects’ motion threshold with a relative probability of detecting motion at 50% due to application of the 1 Hz sinusoidal electrical stimulation across the vestibular end organs. Bilateral bipolar GVS produces acceleration signal toward the cathode electrode, and body’s response/tilt is in the opposite direction, i.e., toward the anode electrode (Fitzpatrick and Day, 2004). Similar to our design, others have also used electrical stimulation using sinusoidal waveforms in the frequency range of 1–2 Hz to determine the threshold by visually observing the amplitude at which subjects swayed at input frequency (Pavlik et al., 1999; Samoudi et al., 2015). These studies have used subjects’ motion threshold levels as the maximum amplitude limit during the posture task in which stochastic stimuli were used. Also, for threshold determination tests we used sinusoidal electrical stimuli while stochastic signals were used in other studies (Yamamoto et al., 2005; Utz et al., 2011) because the majority of our subjects did not discern the direction of induced motion during the application of stochastic stimulation (during pilot testing for this study). Stochastic nature of electrical stimulation of the vestibular organs may have precluded the possibility to objectively detect sway in one direction or the other. Further, sinusoidal motion are easy to discern and hence can yield responses that can be quantified. One of the limitations of our thresholding technique is that sinusoidal stimulation may be highly predictable, and hence it is possible that reported results were influenced by learning or adaptive physiologic mechanisms. To avoid or reduce that effect, the order of presentation of stimulus amplitude was randomized in this study. Further, in future threshold estimations the fixed 20 s of baseline period should be extended by a variable time to be less predictable.

Aihara et al. (2010) have hypothesized that the optimization of behavioral responses may result from interaction between the externally applied noise to the sensory organs at the periphery and the nominal internal noise present in the CNS. For bidirectional stimuli, threshold can be considered as a measure of noise in the brain, which includes all physiologic sources of variability such as afferent noise, processing noise, etc. (Merfeld, 2011). Thus, if the internal noise level is high, the external noise required to elicit optimal response will be less. However, in our study, the peak amplitude of SVS current for optimal performance of balance function did not show any significant relationship with the amplitude of perceptual threshold levels. This maybe because although the amplitudes of perceptual threshold varied in the range of 355–1405 μA the amplitude of peak external noise for the optimal trial that improved walking stability is in a relatively narrow range of 100–500 μA.

### Mechanism of SR using SVS

Galvanic vestibular stimulation was hypothesized to probably act at the spike trigger zone of vestibular afferents (Goldberg et al., 1982, 1984), rather than causing membrane depolarization: maintained GVS generated a maintained series of action potentials (which adapt) during the DC stimulus (Kim and Curthoys, 2004). However, other studies have also demonstrated that electrical current limited to low frequency of stimulation applied across isolated Type I mammalian VHCs evoked mechanical responses of fast length changes of the cell “neck” (Zenner and Zimmermann, 1991; Zenner et al., 1992). These authors hypothesize that as mammalian VHCs are firmly joined to adjacent structures such responses to imposed electrical fields may result in a change in the rotational mechanical characteristics of the stereocilia that will cause a modification in the hair bundle position and have a subsequent effect on the transduction process transfer function during head acceleration and tilt. Hence, we hypothesize that low levels of SVS may help to improve signal detection and information transfer based on the phenomenon of SR with both these mechanisms at the VHC or spike trigger zones of vestibular afferents. Improving signal detection or enhancing information transfer via the vestibular system using the SR phenomenon is further useful because of the convergence and modulation of its activity via proprioceptive inputs at the vestibular nuclei (Jian et al., 2002). Further the vestibular nuclei projects to many areas in the CNS including the flocculus of the cerebellum, the spinal cord, the oculomotor nuclei, and thalamocortical pathway (Jian et al., 2002; Fitzpatrick and Day, 2004). One of those pathways may be involved in alteration of activity in the substantia-nigra pars reticularis (SNr), as SVS at stimulation magnitudes at or below threshold amplitudes have shown to increase the release of γ-amino-butyric acid (GABA) in hemilesioned PD rat models (Samoudi et al., 2012). SVS can modulate the activity of functional brain networks as measured by EEG signals that was demonstrated even after 20–25 s after cessation of a 72 s stimulus (Kim et al., 2013). Thus, SVS at low stimulation magnitudes at or below threshold amplitudes may help improve postural control and stability by facilitating the vestibulo-spinal control system or other non-dopaminergic pathways as shown in PD patients and normal subjects (Pal et al., 2009; Mulavara et al., 2011; Samoudi et al., 2015).

### Usefulness of SVS as an Adjunct to Training

Sensory input from the visual, vestibular, and somatosensory systems are integrated within the CNS to modify appropriate motor output in order to maintain balance for standing or walking. These sensory inputs have pathways that converge at various points in the CNS such as in the spinal cord, vestibular nuclei, thalamus, cortex, and cerebellum that allows for integration of information (Rubin et al., 1979; Aeillo et al., 1983; Wilson, 1991; Wilson et al., 1995; Horak and Hlavacka, 2001). Previous studies have demonstrated that information from one or more sensory information can compensate for faulty or abnormal information from any one of the sensory systems to maintain posture and function (Putkonen et al., 1977; Bles et al., 1984; Dieringer et al., 1984; Inglis et al., 1995; Dieringer, 1997; Sadeghi et al., 2011, 2012; Mulavara et al., 2012; Jamali et al., 2014). This compensation is never complete and these subjects can benefit from additional relevant information that may be provided through the sensory input channels that were deemed inappropriate to facilitate a robust recovery in function. Such examples of compensation are evident in astronauts after their return from spaceflight during their re-adaptation to Earth’s gravity environment. Numerous studies have reported that adaptation to microgravity resulted in the lack of postural control when tested post-flight under conditions that required accurate feedback from the vestibular and ankle proprioception inputs (Paloski et al., 1992, 1994; Paloski, 1998). Another study investigating altered head movement control using a locomotion paradigm showed that exposure to the microgravity environment of spaceflight can cause the central adaptation of the converging vestibular and body load sensing somatosensory inputs (Mulavara et al., 2012). A number of studies have reported that these spaceflight subjects had increased their reliance on visual feedback during their recovery process as a result of adaptation to microgravity (Reschke et al., 1998). Other studies have shown that subjects who rely more on vision for control of movement have more difficulty adapting their walking and postural control strategies to novel sensorimotor discordant environments (Hodgson et al., 2010; Brady et al., 2012; Eikema et al., 2013). However, reports on sensorimotor reconditioning after spaceflight suggested that crewmembers could benefit from systematic head movements with slow increments in their movement amplitudes restricting it to the range tolerated by these subjects during re-entry that may help them to adapt their responses to vestibular input in Earth’s gravity environment to greatly facilitate the re-adaptation process (Wood et al., 2011). Further these same authors report that post-flight reconditioning protocols followed by the crewmembers include a series of exercises that has a primary or secondary benefit with respect to vestibular and proprioception function aiding or improving balance recovery (see Table 1 in Wood et al., 2011). Thus, subjects who are more visually dependent can be trained to modulate information from additional sensory inputs such as proprioception and vestibular inputs to adapt better and faster to novel environments. Similar to the crewmembers adapted to the microgravity environment, the elderly or other clinical populations may also benefit from additional relevant information from the vestibular and proprioceptive channels to facilitate their sensorimotor function. SVS could be used as an adjunct to sensorimotor training paradigms for astronauts (Batson et al., 2011) or other training modalities (Cohen et al., 2005) to improve its efficacy by increasing detection of relevant sub-threshold sensory information via the vestibular system. Indeed, few studies in the literature have investigated the usefulness of SR in conjunction with traditional training paradigms to improve performance (Ross and Guskiewicz, 2006; Ross, 2007; Ross et al., 2007, 2013). These investigators have shown significant improvement in postural balance control aiding recovery when electrical or mechanical SR stimulation to the muscles across the ankle joints was given in conjunction with conventional coordination training compared to training alone (Ross and Guskiewicz, 2006; Ross, 2007; Ross et al., 2007, 2013). Therefore, in general we hypothesize that an individualized sensorimotor training program in conjunction with SVS designed to promote the use of multiple sensory modalities can enhance the ability to adapt postural control and walking stability when exposed to a novel discordant sensory environment in the astronaut, the elderly or other clinical populations.

## Conclusion

The overall objective of this study was to develop a countermeasure based on imperceptible stimulation of the vestibular system with low-level electrical stochastic noise to enhance postural control and walking stability to facilitate adaptation to novel discordant sensory environments. We hypothesized that the detection of time-critical sub-threshold sensory signals using low levels of bipolar SVS based on SR principles will help improve stability of walking during support surface perturbations. This study shows the efficacy of imperceptible stochastic stimulation of the vestibular system based on the SR phenomenon in improving walking stability at an non-zero peak level of stimulation in healthy subjects. This study extends the results of our previous study showing SVS improved postural control in healthy subjects based on the SR phenomenon. Thus, a SR based vestibular stimulation device may be used to improve postural control and walking stability in astronauts, older adults, PD patients with balance problems, patients with either diabetic neuropathy or stroke.

## Conflict of Interest Statement

The authors declare that the research was conducted in the absence of any commercial or financial relationships that could be construed as a potential conflict of interest.
